# Investigating Metrics of Discrete-Individual Repeatability of the Stress Response

**DOI:** 10.1093/iob/obaf005

**Published:** 2025-02-24

**Authors:** U K Beattie, L M Romero, J M Reed

**Affiliations:** Department of Biology, Tufts University, 200 College Ave, Medford, MA 02155, USA; Department of Biology, Tufts University, 200 College Ave, Medford, MA 02155, USA; Department of Biology, Tufts University, 200 College Ave, Medford, MA 02155, USA

## Abstract

There is currently no consensus on the most biologically meaningful way to calculate discrete-individual repeatability of stress response curves. In the current study, we compared three metrics of discrete-individual repeatability that incorporate the whole stress response curve: profile repeatability, Kullback–Leibler (KL) divergence, and hypothalamic–pituitary–adrenal (HPA) flexibility. As part of this work, we present a new R package for computing profile repeatability, “profrep.” Using three datasets (one synthetic and two corticosterone datasets from live birds), our objectives were (1) to compare how these metrics correlate with one another and (2) to determine how representative repeatability scores of fewer replicates were to the “consensus” score (i.e., the score of the full dataset). We found that (1) these three discrete-individual repeatability metrics do not consistently correlate with one another; (2) KL divergence and HPA flexibility are poor at distinguishing individuals from each other (i.e., they give similar scores for each individual regardless of perceived repeatability); and (3) profile repeatability tends to overestimate repeatability when fewer replicates are available, and the consensus score is low. Despite this drawback of profile repeatability, we suggest that it may be the most well-suited metric for assessing discrete-individual repeatability.

## Introduction

A common assumption that is made in the field of organismal biology is that a given sample (or series of samples) is representative of the animal's physiology. In clinical situations, a single measure of a hormone titer is not considered diagnostic. It is why clinicians make multiple measurements of hormone titers before diagnosing a problem, so that if the hormone is outside the normal range, it must be repeatably outside of that range. Additionally, simply relying on summary statistics, such as the mean or median, in an analysis may cause important information to be lost (Williams 2008). Responses such as the stress response, where an entire series of samples (baseline, stress induced, and negative feedback; Dickens and Romero 2013; Romero and Wingfield 2016; Vitousek et al. 2019; Gormally and Romero 2020; Bókony et al. 2021) is the unit being repeated over time, make assessing repeatability/variability more complicated. Not only do we not know how repeatable multipoint samples like a stress response curve are, but there is also not a consensus on how to best quantify that repeatability of an individual, or within-individual variance (Westneat et al. 2015). Although several excellent recent meta-analyses have focused on the repeatability of aspects of the stress response (e.g., Schoenemann and Bonier 2018; Taff et al. 2018; Fanson and Biro 2019), none have specifically addressed the repeatability of the whole stress response curve.

Quantifying repeatability of aspects of the stress response using linear mixed models (LMM repeatability; Dingemanse and Dochtermann 2013; Baugh et al. 2014), especially using the rptR package (Stoffel et al. 2017), seems to be the most popular approach; however, it may not be the best suited for assessing a curve over time (Reed et al. 2019). The purpose of our study was to compare three metrics for assessing discrete-individual repeatability of stress response curves. Profile repeatability (PR) was specifically designed for assessing repeatability of stress response curves and incorporates the variance across replicates at each timepoint and the number of times the replicate lines cross each other (Reed et al. 2019). Kullback–Leibler divergence (KL divergence) is typically used for comparing probability distributions by calculating the probability of predicting one probability distribution given another (Joyce 2011) and takes into consideration the order of replicates. Hypothalamic–pituitary–adrenal flexibility (HPA flexibility) uses the underlying math of heart rate variability, the root mean squares of successive differences (RMSSD), but has been proposed for use with stress response data (Zimmer et al. 2021; Martin and Zimmer 2022). HPA flexibility technically measures how “flexible” or variable a set of replicate stress response curves are; however, we are comparing it to the other two metrics by assuming the opposite of flexibility/variability is repeatability.

Using three datasets, one synthetic dataset and two datasets of stress response curves from living birds, our first goal was to determine the degree to which these repeatability metrics correlate with each other—do they tell the same story? Our second goal was to determine the degree to which repeatability scores calculated from lower numbers of replicate stress response curves were representative of the “consensus” repeatability score (i.e., the repeatability score calculated from a dataset with more replicate samples). Since many repeatability studies in this field are done from a small number of replicates per individual, this will assess the potential bias of having few replicates. We hope these comparisons help stress physiologists make more informed decisions when choosing a discrete-individual repeatability metric to use in their studies.

## Methods

### Datasets

**Fig. 1.**
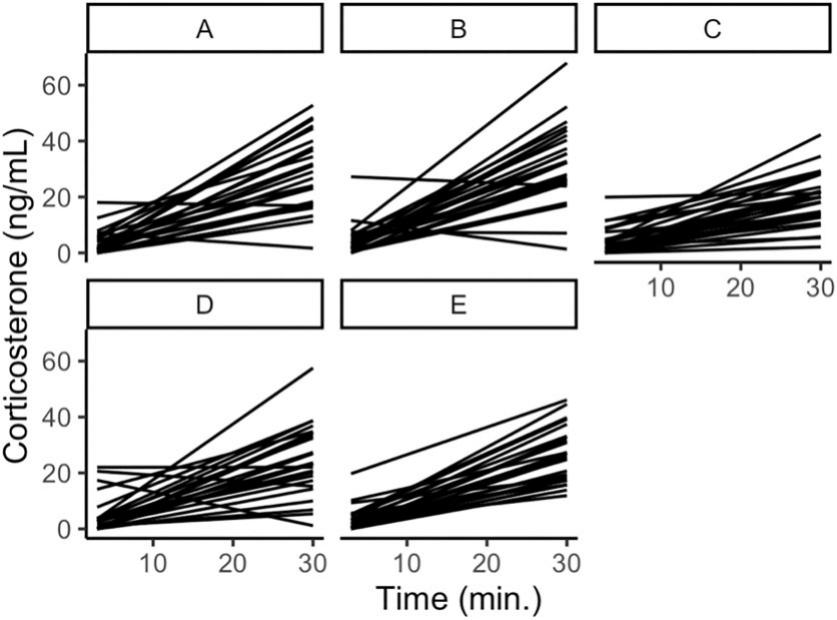
Two-point data. House sparrows were bled at baseline (0 min) and after 30 min of restraint. Each panel represents an individual bird and each line represents one of the 28 replicate stress response curves. Data were extracted from Romero and Rich (2007).

**Fig. 2.**
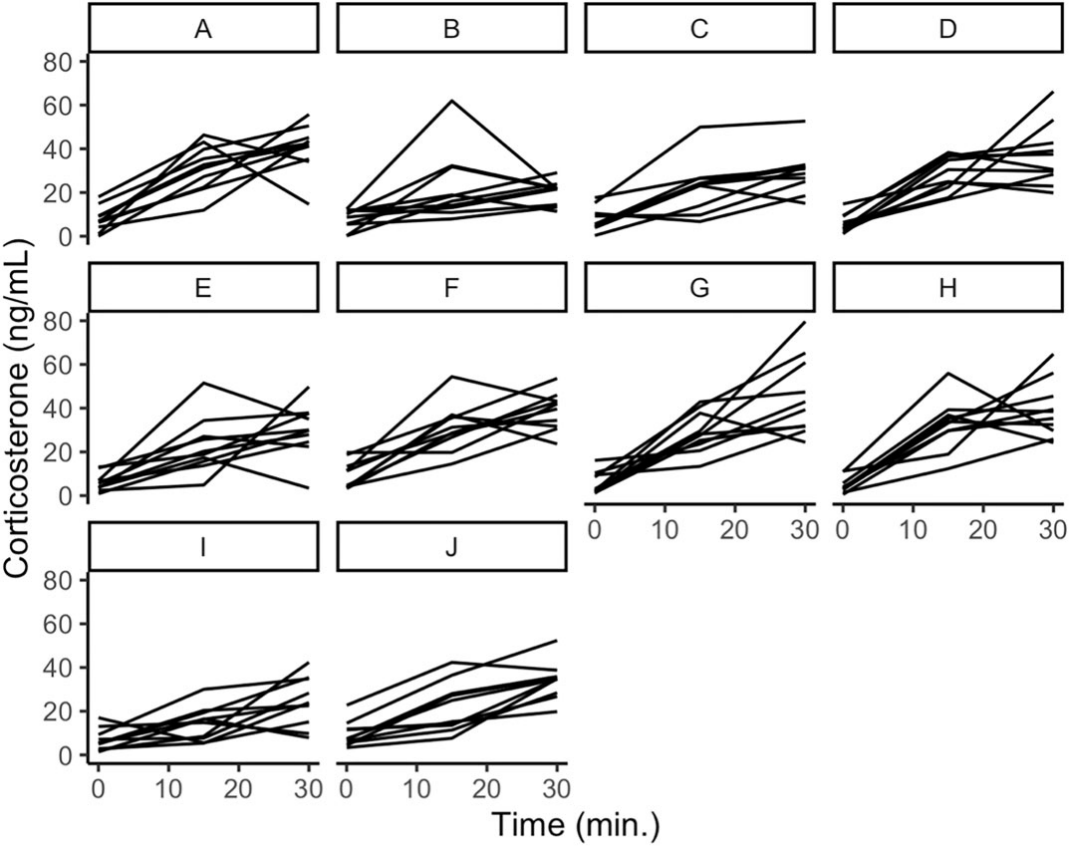
Three-point data. House sparrows were bled at baseline (0 min), after 15 min of restraint, and after 30 min of restraint. Each panel represents an individual bird and each line represents one of the 10 stress response curves. Data were extracted from Rich and Romero (2001).

We analyzed repeatability in three datasets. The first dataset is synthetic data that had been designed to span a range of human-perceived repeatability (taken from Reed et al. 2019). The dataset included 11 animals with four replicate stress response curves of four timepoints. Though this dataset is published in the paper introducing PR (Reed et al. 2019), it was conceived of before the PR metric and then used to inform the development of the metric. The other two datasets were corticosterone data collected from house sparrow (*Passer domesticus*) plasma, one of which has five animals with 28 replicate stress response curves of two timepoints (“two-point data”; Fig. 1; data extracted from Romero and Rich 2007) and the other of which has 10 animals with 10 replicate stress response curves of three timepoints (“three-point data”; Fig. 2; data extracted from Rich and Romero 2001). All three of these datasets are complete because KL divergence (see later) cannot handle missing datapoints.

### Repeatability calculations

For each dataset, we computed three discrete-individual repeatability metrics (PR, KL divergence, and HPA flexibility), and had unbiased observers visually score repeatability. Scorers did not know the background of the project. The visual scoring was done once for each dataset (Reed et al. 2019). Every scorer had training in biology, but varied by experience level (undergraduates, graduate students, one post doc, faculty, and one physician). Scorers were told to rank individuals by degree of repeatability and that ties were allowed. Importantly, we did not provide a definition of repeatability or tell scorers what to look for when assessing repeatability. For the synthetic data, 75% of scorers scored the repeatability of the synthetic individuals in nearly the same order, which we called “the majority rank,” while the remaining 25% of scorers had a ranking that was different from the majority rank, but consistent, so we called this ranking “the minority rank.” For the two-point and three-point data, only the majority rank was used as the minority ranks were inconsistent.

We calculated KL divergence (Joyce 2011) on our time series data without converting to a probability distribution. KL divergence is also known as relative entropy, such that the more different two curves are, the more disordered they are. Applications in biology include comparing amino acid sequences (Akhter et al. 2017) and comparing sleep–wake patterns (Liu et al. 2023). Our code for calculating KL divergence can be found in the supplemental materials. A KL divergence score of 0 means high repeatability and there is no bound for lower repeatability.

HPA flexibility was calculated using the RMSSD following Zimmer et al. (2021) and using the “rmssd_id” function in the “varian” package (Wiley 2016). RMSSD is typically used to quantify heart rate variability, though this metric has been previously published for use with stress response curves (Zimmer et al. 2021; Martin and Zimmer 2022). The lower the RMSSD, the less flexible (or variable) the individual is, so for the purposes of calculating repeatability, the lower the RMSSD, the more repeatable the individual is. As with KL divergence, there is no bound of lower repeatability.

To calculate PR, we developed a new package for R, “profrep” (Beattie et al. 2023) (https://ubeattie.github.io/profrep/) following the original code of Reed et al. (2019). During development and testing of the package, we discovered that the original code contained a bug while filling in missing replicates. The cause of the bug was due to an edge case: when the very first replicate was missing, the original code interpolated the missing value from the “time” column and the next present replicate, rather than the intended interpolation between the value for the last and next present replicate. The “profrep” package presented here fixes this bug. We have verified that this bug does not affect the results of Reed et al. (2019) as this specific edge case was not present in the original data. To confirm the accuracy of this package compared to the original code, we checked the results using the three datasets in the present study, one of which was taken from Reed et al. (2019). The “profrep” package can be installed directly from CRAN (https://cran.r-project.org/package=profrep) by running “install.packages (‘profrep’).” Note that for PR, a score of 0 means low repeatability, while a score of 1 means high repeatability.

### Correlation of repeatability metrics

For each dataset, we computed pairwise correlations between the scorer's rankings and the three discrete-individual repeatability metrics. As described earlier, a score of 1 is highly repeatable for profile repeatable and a score of 0 is highly repeatable for KL divergence and HPA flexibility (low flexibility), so to make the repeatability metrics comparable, we correlated the rankings from most to least repeatable, rather than using the calculated scores. We did this for two reasons: (1) because each metric is calculated on a different scale, comparing scores is problematic; (2) even more importantly, it detracts from evolutionary comparisons since the rank relative to other individuals is what matters, rather than the value per se. A Pearson correlation coefficient was calculated for each pairwise comparison. For the correlations, we only compared rankings of repeatability metrics that had the same number of replicate curves (i.e., every “animal” in the synthetic dataset had 4 replicate curves, every animal in the two-point dataset had 28 replicate curves, and every animal in the three-point dataset had 10 replicate curves, so we computed three sets of pairwise comparisons, one for each dataset separately).

### Combination analysis

Next, we wanted to determine whether, for each repeatability metric, repeatability scores calculated on fewer replicate stress response curves were representative of the “consensus” repeatability score (i.e., the score calculated from the total number of replicates available for each dataset—4 for the synthetic data, 28 for the two-point data, and 10 for the three-point data).

For the synthetic data, we calculated the three discrete-individual repeatability scores (PR, KL divergence, and HPA flexibility) for the six combinations of two replicate curves (i.e., replicate 1 + replicate 2; replicate 1 + replicate 3; etc.) and for the four combinations of three replicate curves, without replacement.

For both sets of the house sparrow data, we did the same type of combinations; 28 pick 2, 28 pick 3 . . . 28 pick 26, 28 pick 27 for the two-point data, or 10 pick 2, 10 pick 3 . . . 10 pick 8, 10 pick 9 for the three-point data. For some of the number of replicates, however, there would have been over a million combinations. When there were over 100 possible combinations, we randomly selected 100 upon which to compute the repeatability scores; when there were under 100 possible combinations, we computed the repeatability scores on every possible combination. We then graphed boxplots of the repeatability scores, with each panel representing an individual in the order of the “consensus” repeatability score.

## Results

All discrete-individual repeatability scores and rankings can be found in Table 1.

**Table 1.** Repeatability scores and rankings for all three datasets

				Profile repeatability		KL divergence		HPA flexibility	
Dataset	Bird	Majority Rank	Minority Rank	Score	Rank	Score	Rank	Score	Rank
Synthetic	E	1	8	0.99	1	0.61	1	19.13	6
	B	2	2	0.99	2	2.55	2	14.52	2
	D	3	6	0.99	3	3.50	3	31.54	11
	F	4	4	0.98	4	30.45	11	18.21	5
	G	5	3	0.96	5	19.27	8	31.06	10
	I	6	1	0.95	6	15.84	5	16.56	3
	C	7	4	0.89	8	15.45	4	13.67	1
	H	8	7	0.44	10	24.06	10	20.39	7
	A	9	8	0.20	11	21.67	9	18.08	4
	J	10	10	0.90	7	15.84	5	21.88	8
	K	11	11	0.76	9	15.84	5	23.93	9
Two-point	C	1		0.96	1	14.10	2	17.33	1
	E	2		0.94	2	9.42	1	24.31	3
	A	3		0.70	4	15.31	3	27.21	4
	B	4		0.53	5	15.89	4	31.05	5
	D	5		0.81	3	20.57	5	24.27	2
Three-point	H	1		0.63	7	11.80	4	29.63	9
	F	2		0.87	2	10.44	1	24.46	7
	C	3		0.86	3	14.84	6	19.02	3
	E	4		0.71	6	18.84	10	22.26	5
	I	5		0.85	4	14.18	5	17.08	1
	A	6		0.84	5	16.61	8	27.78	8
	J	7		0.89	1	11.60	3	20.09	4
	B	8		0.34	9	18.01	9	17.64	2
	G	9		0.05	10	15.03	7	32.60	10
	D	10		0.50	8	10.57	2	24.44	6

For profile repeatability, a score of 0 is not repeatable and a score of 1 is highly repeatable. For KL divergence and HPA flexibility, a score of 0 is highly repeatable and there is no limit for low repeatability (higher score = lower repeatability). Within each dataset, birds are in order of the majority rank from human observers.

**Fig. 3.**
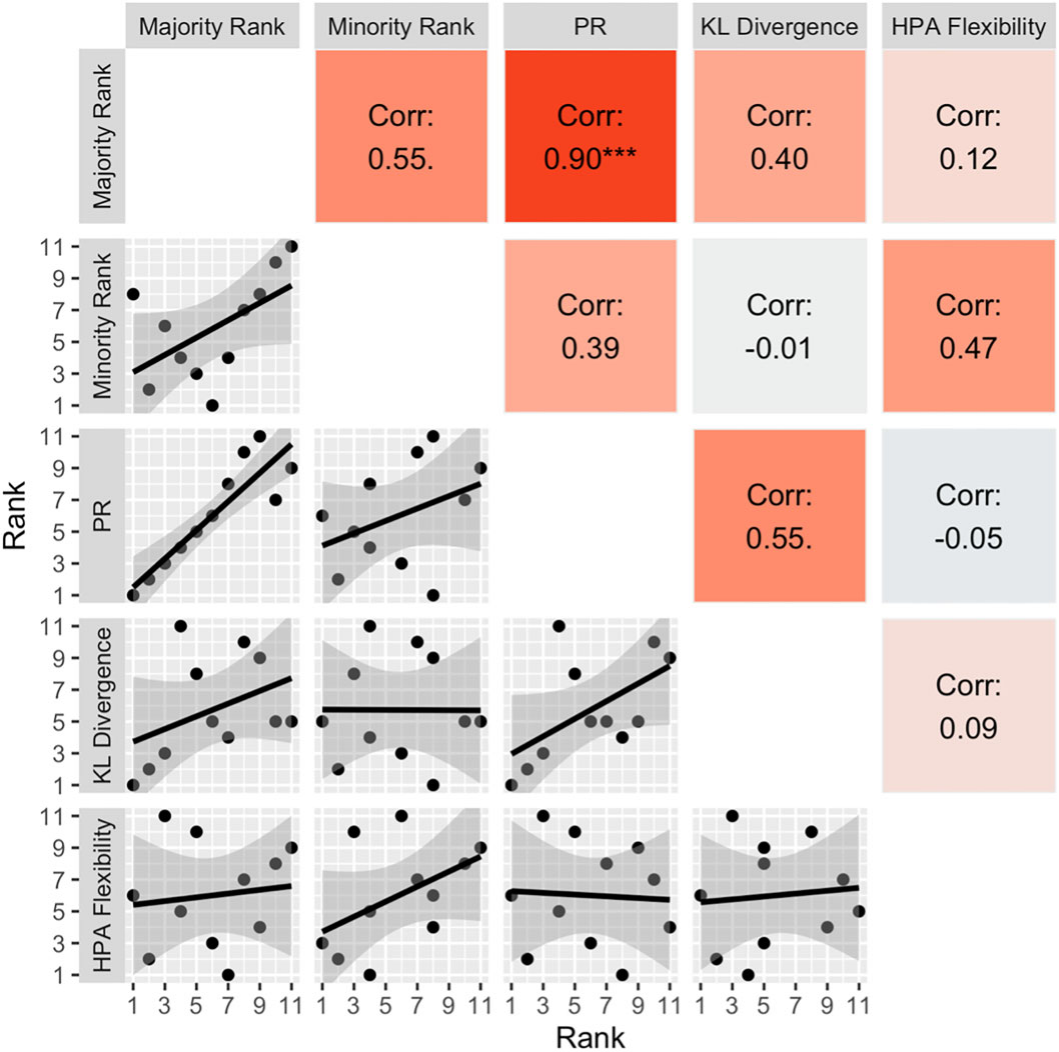
Correlation matrix of rankings from three repeatability metrics and people's visual repeatability rankings from the synthetic data. A ranking of 1 is the most repeatable (or least flexible). PR = profile repeatability; KL divergence = Kullback–Leibler divergence; Corr = Pearson correlation coefficient (*r*). “***” denotes a correlation coefficient with a *P*-value < 0.001; “*” < 0.05; “.” denotes *P* < 0.10.

**Fig. 4.**
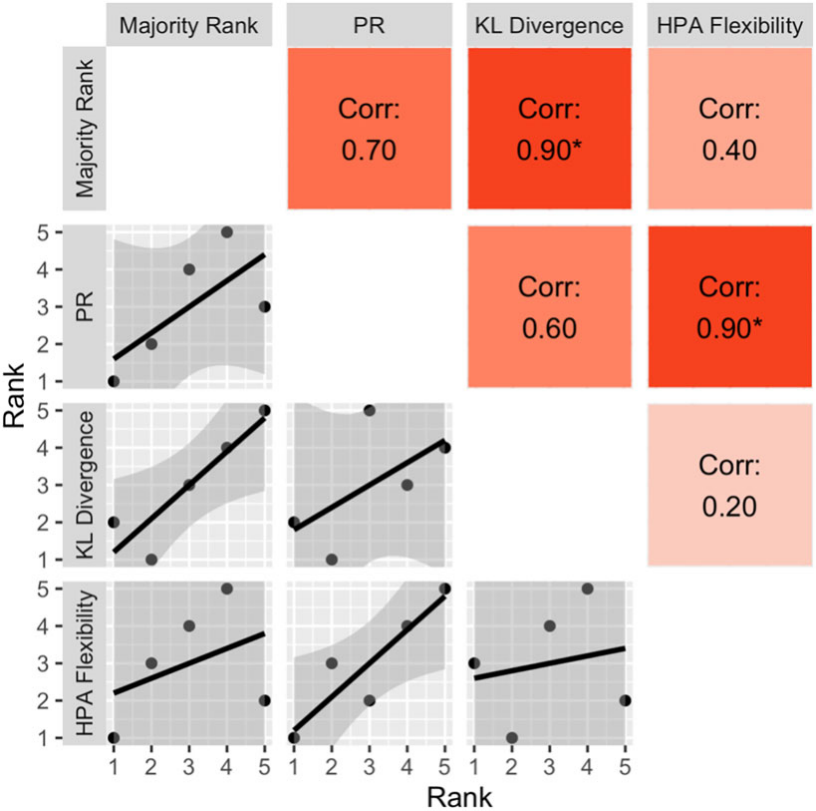
Correlation matrix of rankings from three repeatability metrics and people's visual repeatability rankings from the two-point data. A ranking of 1 is the most repeatable (or least flexible). PR = profile repeatability; KL divergence = Kullback–Leibler divergence; Corr = Pearson correlation coefficient (*r*). “*” < 0.05.

**Fig. 5.**
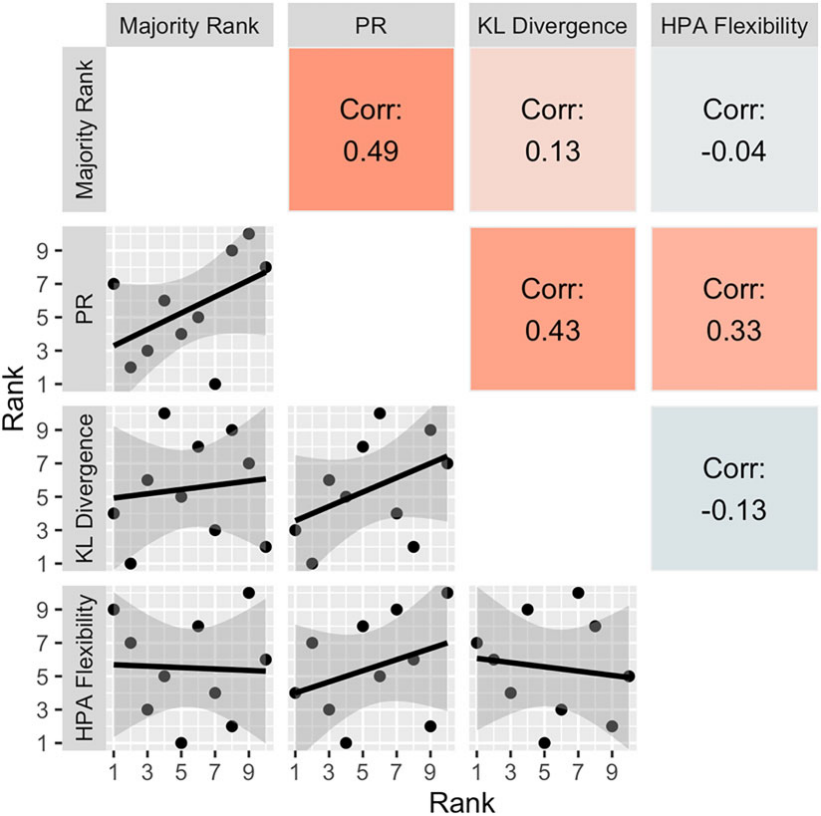
Correlation matrix of rankings from three repeatability metrics and people's visual repeatability rankings from the three-point data. A ranking of 1 is the most repeatable (or least flexible). PR = profile repeatability; KL divergence = Kullback–Leibler divergence; Corr = Pearson correlation coefficient (*r*).

For all of the correlation analyses, there were no statistically significant consistent correlations between any pair of metrics across all comparisons (i.e., the results of the pairwise correlations depended on the dataset being analyzed). Specifically, within the synthetic data (Fig. 3), most associations tended to be positive, and there was a statistically significant correlation only between PR and the majority rank (*r* = 0.90). For the two-point data (Fig. 4), associations again tended to be positive, and statistically significant correlations occurred only between PR and HPA flexibility (*r* = 0.90), and KL divergence and the majority rank (*r* = 0.90). No correlations were statistically significant for the three-point data, and some relationships tended negative (Fig. 5).

**Fig. 6.**
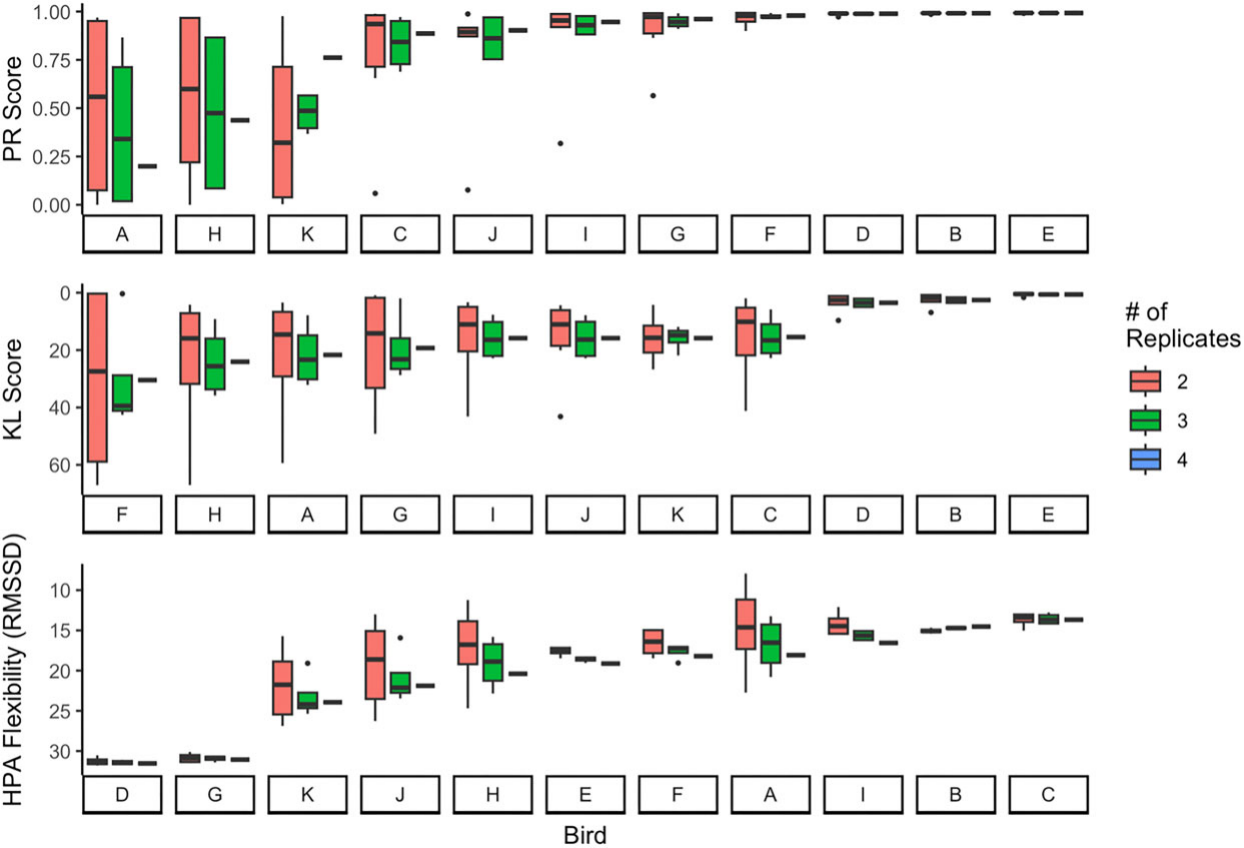
Combination analysis for synthetic data. For each bird, we computed three repeatability metrics for every possible combination of number of replicates, given the four to choose from. Birds on the *x*-axis are in the order of least repeatable (left) to most repeatable (right) according to their “consensus” repeatability score (score calculated on all four replicate curves). Note that the order is different for the three panels and that higher on the *y*-axis means higher repeatability.

**Fig. 7.**
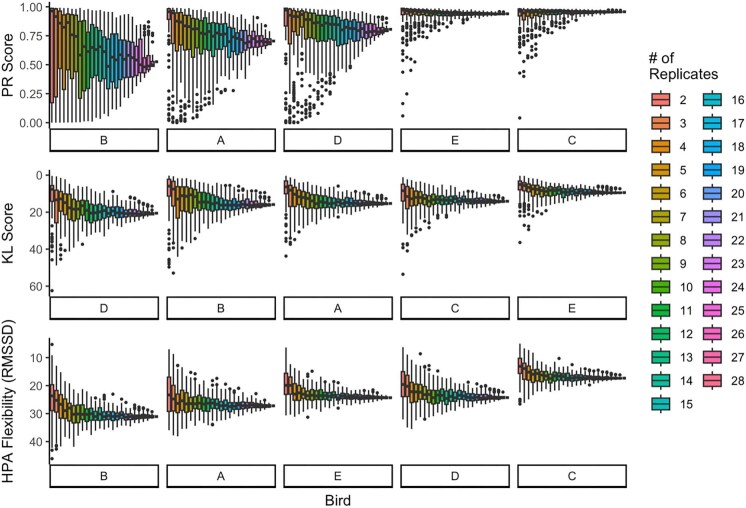
Combination analysis for the two-point data. For each bird, we computed three repeatability metrics for 100 possible combinations of number of replicates, given the 28 to choose from. Birds on the *x*-axis are in the order of least repeatable (left) to most repeatable (right) according to their “consensus” repeatability score (score calculated on all 28 replicate curves). Note that the order is different for the three panels and that higher on the *y*-axis means higher repeatability.

**Fig. 8.**
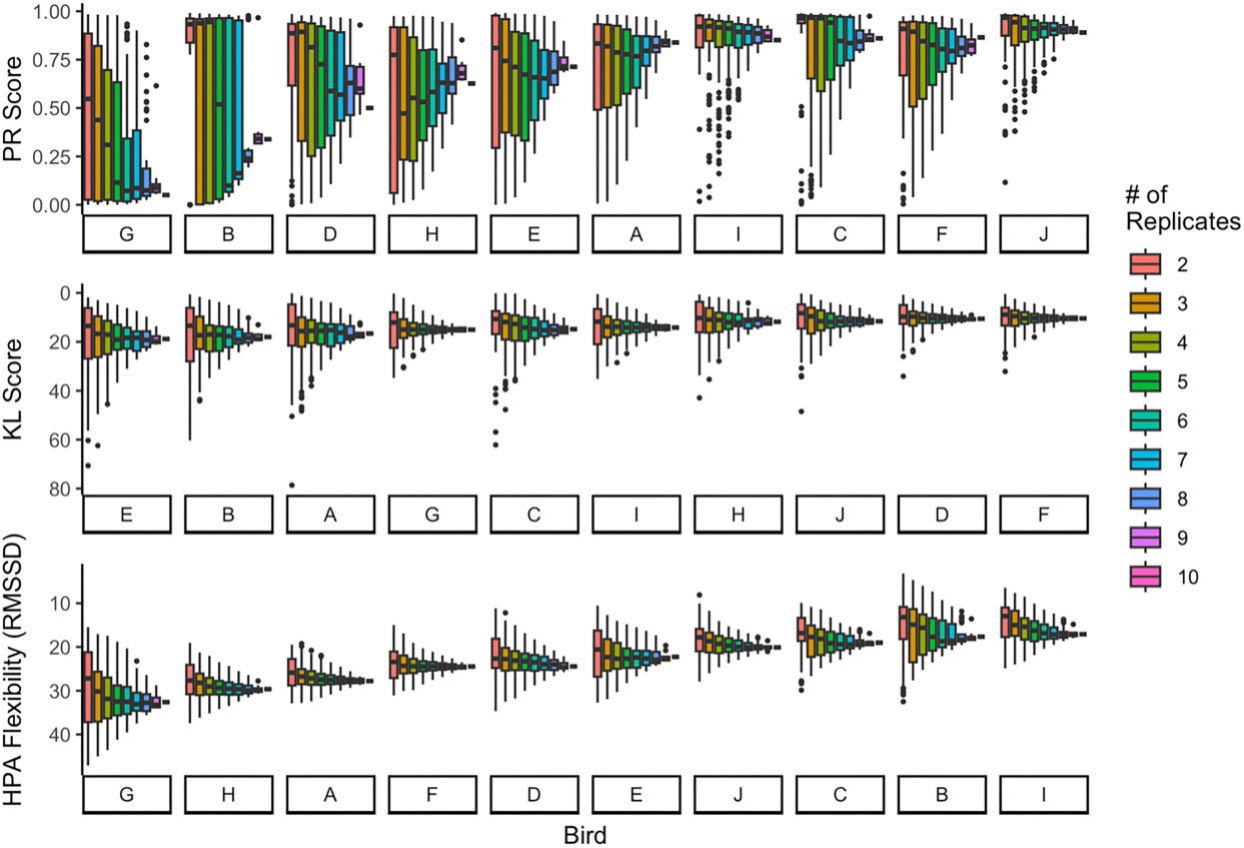
Combination analysis for the three-point data. For each bird, we computed three repeatability metrics for 100 possible combinations of number of replicates, given the 10 to choose from. Birds on the *x*-axis are in the order of least repeatable (left) to most repeatable (right) according to their “consensus” repeatability score (score calculated on all 10 replicate curves). Note that the order is different for the three panels and that higher on the *y*-axis means higher repeatability.

Combination analyses showed that the reliability of lower replicate numbers depended on the repeatability metric used. We found that variances in all repeatability scores tended to increase as the number of replicates per individual decreased. Across all metrics, the median values of scores calculated on fewer replicates tended to overestimate repeatability (Figs. 6–8). This finding was particularly evident in PR scores when the consensus score was low (but when the consensus score was high, fewer replicates were representative; Figs. 6–8, top panels) and was less extreme in KL scores (Figs. 6–8, middle panels).

In addition, we note that KL divergence and HPA flexibility both show little variation across individuals in repeatability scores in the bird datasets, though the same is not true for the synthetic data. In contrast, PR scores show extensive variation between individuals in repeatability.

## Discussion

Regarding the concept of repeatability, evolutionary biologists and physiologists have been discussing two different, yet related, concepts. The repeatability that has most often been measured in the evolution literature is a population-level measurement used to understand trait heritability and evolution (although see Westneat et al. 2015). This analysis relies upon the measurement of individual phenotypes in the population, but what is the stress hormone phenotype of an individual? The assumption of a population-level analysis seems to be that the typically single measurement of the hormone is the phenotype of that individual, which can then be fed into the models to determine the population-level variation. However, as physiologists, we must ask whether that measurement does, in fact, represent the actual phenotype of the individual. If the hormone titer varies every time it is measured, what is the phenotype? What is selection acting on? We believe that if the hormone titer is different every time it is measured (i.e., not repeatable), then one cannot determine heritability or changes in allelic frequencies. This concept is what physiologists are defining as repeatability. Although a recent statistical technique shows how one can calculate within-individual variance (Westneat et al. 2015), this technique has not (yet) been widely adopted.

The goal of the present study was to compare and evaluate methods of computing repeatability in the stress response curve of discrete individuals: PR, KL divergence, and HPA flexibility. These metrics indicated that the population within each dataset we analyzed is comprised of both individuals with low repeatability and high repeatability, as well as individuals in the middle. This information is not captured when assessing repeatability only at a population level.

Unfortunately, none of the three discrete-individual repeatability metrics that we compared were perfect. One option for assessing a response profile on an individual basis is KL divergence; however, this metric is based on the area under the curve (Joyce 2011). This is problematic because two stress response profiles could have the same area under the curve, but completely opposite slopes (such as −1 and 1 between two time points) and therefore, potentially different biological consequences (Lattin et al. 2011). Similarly, depending on the intercept, a flat line (slope of 0) could also have the same area under the curve and the previous two hypothetical responses with slopes of 1 and −1. These examples are of straight lines for simplicity, but the same problem could occur with a three-point (or more) stress response curve. There are many potential stress response curves that could have very different fitness consequences but would be indiscernible from calculating area under the curve. Additionally, KL divergence cannot handle missing datapoints, a situation that is not uncommon in stress physiology. A solution could be to fill the missing point using interpolation, but this would artificially increase calculated repeatability (Reed et al. 2019), a consequence that is corrected for in the calculation of PR.

Two metrics that have been proposed specifically for use with stress response curves are HPA flexibility (Zimmer et al. 2021; Martin and Zimmer 2022) and PR (Reed et al. 2019). HPA flexibility is calculated using the RMSSD, which is typically calculated for assessing heart rate variability. There are two concerns with HPA flexibility—the first is that collapsing an entire stress response curve into an average loses a lot of information. Just as with KL divergence, there are many different potential stress response curves that could have the same average value. The second concern is that replicate order (i.e., which within-individual replicate value is used first) is important in the calculation, but it is not clear why if it is always biologically meaningful for stress response curves, like it is for heart rate variability. For example, stressors with low severity that reoccur after sufficient recovery are normally thought to elicit equivalent responses. Although heart beats are biologically successive, successive sampled stress response curves might be successive only because the experimenter chose to take blood samples at those times. There could have been stressors (which caused stress responses) between “successive” sampled stress response curves, which would be missing from the data. While it is true that some stress responses may be altered by prior stress responses, samples taken weeks or months apart should not necessarily have order weighted as strongly. Additionally, a recent metareview (Taff et al. 2018) noted that repeatability, using the LMM method, decreased with increased time between sampling, and using HPA flexibility may compound this effect.

Lastly, PR uses the variances at each timepoint, the maximum variance, the number of crossings (lines that cross over each other), and the number of replicates to compute the repeatability score (Reed et al. 2019). PR was specifically designed for assessing the repeatability of stress responses by incorporating the shape of the response curves. By using PR, which incorporates both baseline (which bind preferentially to mineralocorticoid receptor) and stress-induced concentrations (which bind preferentially to glucocorticoid receptor) in the single metric, it can capture the nuance of switching from mineralocorticoid receptor to glucocorticoid receptor binding (Lattin et al. 2012) and fits with previously published models of stress (McEwen and Wingfield 2003; Romero et al. 2009). However, these advantages are also the target of criticism—that this metric is ad hoc and could potentially be less generalizable. Reed et al. (2019) intended their metric to be used as a starting point for statisticians to create a more generalizable metric for assessing repeatability of curves.

### Discrimination of repeatable individuals versus not-repeatable individuals

The range of potential repeatability scores is 0 (least repeatable) to 1 (most repeatable) for PR, and infinity (least repeatable) to 0 (most repeatable) for KL divergence and HPA flexibility. This means that KL divergence and HPA flexibility do not have a numerical limit for lower repeatability, which makes interpretation of the scores difficult. For example, individuals in the synthetic dataset and the three-point dataset span almost the whole range of potential PR scores (0.20–0.99 for the synthetic data and 0.05–0.89 for the three-point data; Table 2). In contrast, the birds in the two-point dataset only span PR scores of 0.53–0.96, and because of the lower and upper bounds of the score, we can be confident that none of the birds had low repeatability. Additionally, PR has the most discrimination in the middle of the distribution, giving further context to the scores themselves (Reed et al. 2019). For KL divergence, it is not clear how meaningful the difference between a score of 0.61 and 30.45 is (the KL scores for the most repeatable and least repeatable of the synthetic data, respectively; Table 2) when there is no context for what constitutes low repeatability. The same is true for HPA flexibility—the HPA flexibility scores in our study ranged between 13.67 and 31.54 for the synthetic data, but how to interpret the magnitude of the difference is not clear. There is relatively little variation in the HPA flexibility and KL divergence scores across individuals and this is especially true in the wild bird datasets. For example, in the two-point data, PR indicates that there are two birds that are highly repeatable and three that are mid-level repeatable, whereas, according to KL divergence and HPA flexibility, their scores are nearly identical to the point that even the order of the birds is slightly different. In sum, while relative comparisons can be made with HPA flexibility and KL divergence, the bounded nature of PR gives us a stronger basis on which to compare differences in scores.

**Table 2.** Summary of findings and suggestions for researchers

	Profile repeatability	KL divergence	HPA flexibility
Output range (not repeatable to repeatable)	0–1	∞–0	∞–0
Average individual repeatability	✓	✓	✓
Discrete-individual repeatability	✓	✓	✓
Robust to low sample size		✓	✓
Discrimination ability	✓		
Context for degree of repeatability	✓		
Correlation to majority ranking	✓✓✓	✓✓	✓
Correlation to minority ranking	✓✓		✓✓

Note that multiple check marks in the last two rows refer to the Pearson correlation coefficient for the synthetic data (no check indicates *R* < 0; ✓ indicates 0 < *R* < 0.33; ✓✓ indicates 0.33 *R* < 0.66; ✓✓✓ indicates 0.66 < *R* < 1).

### Correlations

The strongest rank correlation of discrete-individual repeatability scores was between the majority rank (a visual assessment by multiple people) and PR in the synthetic data, which was expected as PR was derived based on traits from the majority rank. While there were other statistically significant correlations, none were consistent across datasets (e.g., PR and majority rank were not significantly correlated in every dataset). Conversely, there are two negative (not statistically significant) relationships in the three-point data: between HPA flexibility and majority rank, and HPA flexibility and KL divergence. The correlations (or lack thereof) indicate that the reliability of these individual repeatability metrics is very dependent on the data being assessed. Greater sample sizes would be needed to clarify the relationship between these metrics. Alternatively, and/or in addition, the lack of correlations may point to each of these metrics measuring different aspects of repeatability (although what those differences might be is not presently clear), in which case high correlations would be unexpected.

### Representativeness of low replicate number

One goal of this paper was to determine whether each discrete-individual repeatability metric had a minimum number of replicates required to reliably detect the consensus repeatability score (i.e., the score calculated using all available replicates). Overall, the fewer timepoints taken, the more replicate stress response curves were needed to feel confident in the repeatability metric. Of course, two points make a line, not a curve, but we included two-point data here because so many studies only take two points. This paper presents data from house sparrows; however, the actual numbers of replicate stress response curves needed for a consensus value may need to be validated by species. We found that with fewer replicates, as is common in stress physiology, a researcher is more likely to calculate a higher PR score than might be “true.” Because it seems to be more sensitive to outliers, we recommend limiting calculating PR to situations with at least 7 replicate stress response curves with three timepoints or 15 replicate stress response curves with two timepoints. We recognize, however, that this is likely to be difficult to achieve in field studies. KL divergence and HPA flexibility calculated on fewer replicates seem to more closely match the consensus scores—for data with three timepoints, we recommend at least 5 replicate stress response curves, and for data with two timepoints, we recommend at least 8 replicate stress response curves. Although KL divergence and HPA flexibility require less data to calculate a consistent estimate of repeatability, as stated earlier, neither method is good at distinguishing between individuals of high and low repeatability for two reasons—there is little variability in HPA flexibility or KL divergence scores and neither method has bounded values as discussed earlier.

### Conclusions and suggestions for researchers

While all of the repeatability metrics assessed in this paper have their strengths and weaknesses, we believe that some may be better suited for stress physiology than others (Table 2). Researchers should note that KL divergence and HPA flexibility show little discrimination among individuals in the bird datasets, while PR has both context for low and high (0–1, respectively) as well as high discrimination in the middle of the distribution (Reed et al. 2019). We suggest that stress physiologists use KL divergence as a repeatability metric only if they are interested in comparing the repeatability of total glucocorticoid exposure over time, as would be assessed by the area under a response curve over time. Similarly, we suggest that stress physiologists use HPA flexibility only if there is a biological relevance of the collection order of the stress response curves. However, researchers should still take caution in reducing a stress response curve to the average value as is done in HPA flexibility. Despite the ad hoc nature of PR, we believe it is the most well-suited metric currently available to assess repeatability of glucocorticoids in discrete individuals, though researchers should be aware that it may mischaracterize repeatability when a small number of replicate stress response curves are available. Finally, we have published an R package for computing PR, which is now listed in the CRAN directory, to make this metric more readily available to researchers.

## Supplementary Material

obaf005_Supplemental_File

## Data Availability

The data underlying this article will be shared on reasonable request to the corresponding author.
